# Life beyond the Tanimoto coefficient: similarity measures for interaction fingerprints

**DOI:** 10.1186/s13321-018-0302-y

**Published:** 2018-10-04

**Authors:** Anita Rácz, Dávid Bajusz, Károly Héberger

**Affiliations:** 1 Plasma Chemistry Research Group, Research Centre for Natural Sciences, Hungarian Academy of Sciences, Magyar tudósok krt. 2, Budapest, 1117 Hungary; 2 Medicinal Chemistry Research Group, Research Centre for Natural Sciences, Hungarian Academy of Sciences, Magyar tudósok krt. 2, Budapest, 1117 Hungary

**Keywords:** Virtual screening, Interaction fingerprint, Similarity metrics, SRD, ANOVA, FPKit, Binary fingerprints

## Abstract

**Background:**

Interaction fingerprints (IFP) have been repeatedly shown to be valuable tools in virtual screening to identify novel hit compounds that can subsequently be optimized to drug candidates. As a complementary method to ligand docking, IFPs can be applied to quantify the similarity of predicted binding poses to a reference binding pose. For this purpose, a large number of similarity metrics can be applied, and various parameters of the IFPs themselves can be customized. In a large-scale comparison, we have assessed the effect of similarity metrics and IFP configurations to a number of virtual screening scenarios with ten different protein targets and thousands of molecules. Particularly, the effect of considering general interaction definitions (such as Any Contact, Backbone Interaction and Sidechain Interaction), the effect of filtering methods and the different groups of similarity metrics were studied.

**Results:**

The performances were primarily compared based on AUC values, but we have also used the original similarity data for the comparison of similarity metrics with several statistical tests and the novel, robust sum of ranking differences (SRD) algorithm. With SRD, we can evaluate the consistency (or concordance) of the various similarity metrics to an ideal reference metric, which is provided by data fusion from the existing metrics. Different aspects of IFP configurations and similarity metrics were examined based on SRD values with analysis of variance (ANOVA) tests.

**Conclusion:**

A general approach is provided that can be applied for the reliable interpretation and usage of similarity measures with interaction fingerprints. Metrics that are viable alternatives to the commonly used Tanimoto coefficient were identified based on a comparison with an ideal reference metric (consensus). A careful selection of the applied bits (interaction definitions) and IFP filtering rules can improve the results of virtual screening (in terms of their agreement with the consensus metric). The open-source Python package FPKit was introduced for the similarity calculations and IFP filtering; it is available at: https://github.com/davidbajusz/fpkit.
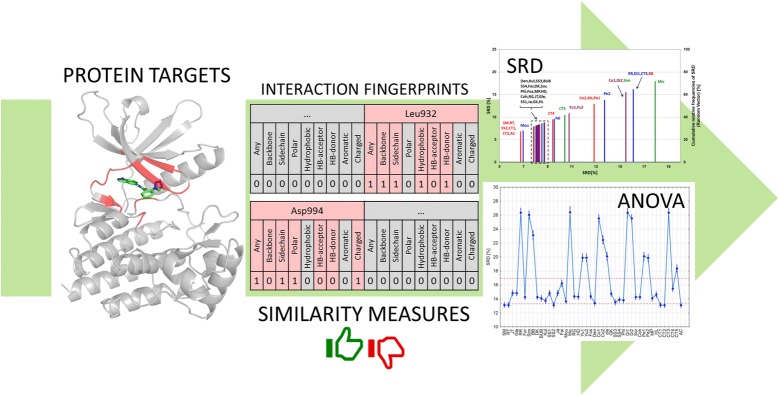

**Electronic supplementary material:**

The online version of this article (10.1186/s13321-018-0302-y) contains supplementary material, which is available to authorized users.

## Introduction

Interaction fingerprints are a relatively new concept in cheminformatics and molecular modeling [1]. As molecular fingerprints are binary (or bitstring) representations of molecular structure, analogously, interaction fingerprints are binary (or bitstring) representations of 3D protein–ligand complexes. Each bit position of an interaction fingerprint corresponds to a specific amino acid of the protein and a specific interaction type. A value of 1 (“on”) denotes that the given interaction is established between the given amino acid and the small-molecule ligand (a 0, or “off” value denotes the lack of that specific interaction). Two such fingerprints are most commonly compared with the Tanimoto similarity metric (taking a value between 0 and 1, with 1 corresponding to identical fingerprints, i.e. protein–ligand interaction patterns). In the most common setting, the Tanimoto similarity is calculated between a reference fingerprint (usually belonging to a known active molecule) and many query fingerprints.

Despite the straightforward definition, interaction fingerprints have been implemented by various research groups and commercial software developers with slight differences in the specifics. The first interaction fingerprint was termed structural interaction fingerprint (SIFt) and was introduced by Deng et al. [2]. This implementation contained originally seven interaction types (any contact, backbone contact, sidechain contact, polar contact, hydrophobic contact, H-bond donor and acceptor), and was later extended to include aromatic and charged interactions as well [3]. This modified version is implemented in the popular Schrödinger molecular modeling suite, which we also applied in this work, see Table 1 [4]. A similar implementation was published by Cao and Wang [5], containing 10 interactions per residue, and termed ligand-based interaction fingerprint (LIFt).

**Table 1:** Summary of the bit definitions of the modified SIFt implemented in the Schrödinger Suite and applied in this work

Abbreviation	Short definition	Description
Any	Any contact	A ligand atom is within the required distance of a receptor atom
BB	Backbone interaction	A ligand atom is within the required distance of a receptor backbone atom
SC	Sidechain interaction	A ligand atom is within the required distance of a receptor side chain atom
Pol	Polar residues	A ligand atom is within the required distance of an atom in a polar residue of the receptor (ARG, ASP, GLU, HIS, ASN, GLN, LYS, SER, THR, ARN, ASH, GLH, HID, HIE, LYN)
Hyd	Hydrophobic residues	A ligand atom is within the required distance of an atom in a hydrophobic residue of the receptor (PHE, LEU, ILE, TYR, TRP, VAL, MET, PRO, CYS, ALA, CYX)
HBA	Hydrogen bond acceptor	The ligand forms a hydrogen bond with an acceptor in a receptor residue
HBD	Hydrogen bond donor	The ligand forms a hydrogen bond with a donor in a receptor residue
Aro	Aromatic residue	A ligand atom is within the required distance of an atom in an aromatic residue of the receptor (PHE, TYR, TRP, TYO)
Chg	Charged residue	A ligand atom is within the required distance of an atom in a charged residue of the receptor (ARG, ASP, GLU, LYS, HIP, CYT, SRO, TYO, THO)

A widely-applied variant, simply termed interaction fingerprint (IFP) was introduced by Marcou and Rognan [6], containing seven interactions per residue. A marked difference between SIFt and IFP is that IFP differentiates aromatic interactions by their orientations (face-to-face vs. edge-to-face), and charged interactions by the specific charge distribution (i.e. cation on the ligand vs. anion on the ligand). Furthermore, IFPs can be configured to include less common interaction types, such as weak H-bonds or cation–π interactions. Later, the same group has introduced triplet interaction fingerprints (TIFPs), which encodes triplets of interaction points to a fixed length of 210 bits [7].

Mpamhanga et al. [8] have introduced three types of interaction fingerprints in their work in 2006, out of which the one termed CHIF is probably the most prominent. Atom-pairs based interaction fingerprint (APIF) is a variant implemented by Pérez-Nueno et al. [9] in the MOE SVL scripting language [10]. APIF accounts for the relative positions of pairs of interactions (based on their binned distances) and stores them in a count-based fingerprint with a fixed length (294 bits).

Da and Kireev [11] have introduced SPLIF (Structural protein–ligand interaction fingerprints), whose main difference with respect to SIFt is that the interactions are encoded only implicitly by encoding the interacting ligand and protein fragments (whereas in SIFt the interaction type explicitly defines the given bit in the bitstring). In the same year, Sato and Hirokawa [12] have introduced another approach called PLIF (protein–ligand interaction fingerprints), which relies on the per-residue identification of the number of interacting atoms (with the ligand). To our knowledge, the most recent novel interaction fingerprint implementation is the PADIF (Protein per atom score contributions derived interaction fingerprint) approach of Jasper et al. [13]. PADIF incorporates the strengths of the different interactions by exploiting the per atom score contributions of the protein atoms, which are calculated for each pose during docking with GOLD, or with any other scoring function that can output atom contributions [14]. As a consequence, PADIF is an atom-based interaction fingerprint.

Interaction fingerprints have been applied numerous times to complement docking scores in virtual screening campaigns, e.g. for the discovery of GPCR (G-protein coupled receptor) ligands [15] or kinase inhibitors [16]. In more complex examples, they have been applied for interpreting activity landscapes [17], for training machine learning models [18], and for identifying covalently targetable cysteine residues in the human kinome [19]. Additionally, interaction fingerprints are applied to support large, specialized structural databases, such as GPCRdb (for GPCRs) [20], KLIFS (for kinases) [21, 22] or PDEstrian (for phosphodiesterases) [23].

Binary similarity measures are applied in various scientific fields to compare binary and continuous data vectors. To our knowledge the most comprehensive collection of similarity measures was published by Todeschini et al. [24], listing 51 similarity measures (out of which seven have been shown to perfectly correlate with others).

For binary data (e.g. for two interaction fingerprints), similarity measures are calculated from the contingency table (or confusion matrix) shown in Table 2, containing the frequencies of four events: (a) 1–1 (interaction present in both complexes), (b) 1–0 (interaction present in the first complex and absent from the second), (c) 0–1 (interaction absent from the first complex but present in the second), and (d) 0–0 (interaction absent from both complexes). With these parameters (along with the fingerprint length *p*), various similarity measures can be calculated, as exemplified here:

1 $$SM = \frac{a + d}{p}$$

2 $$JT = \frac{a}{a + b + c}$$

3 $$BUB = \frac{{\sqrt {ad} + a}}{{\sqrt {ad} + a + b + c}}$$

In the examples, *SM* is the simplest similarity coefficient (called *simple matching*, or *Sokal*–*Michener*), *JT* corresponds to the *Jaccard*–*Tanimoto* coefficient (the de facto standard of the cheminformatics community), and *BUB* is the *Baroni*–*Urbani*–*Buser* coefficient that was suggested in our recent work as a good similarity metric for metabolomics fingerprints [25].

**Table 2:** Confusion matrix for a pair of interaction fingerprints, containing the frequencies of common on bits (*a*), common off bits (*d*), and exclusive on bits for Complex 1 (*b*) and Complex 2 (*c*)

*p *=* a *+* b *+* c *+* d*	Complex 2	
	1 (interaction present)	0 (interaction absent)
*Complex 1*		
1 (interaction present)	*a*	*b*
0 (interaction absent)	*c*	*d*

The values of similarity measures usually range from 0 to 1 (as for the above examples), but many of them (e.g. correlation-based measures) are defined to other ranges, such as − 1 to + 1. Such measures can be rescaled to the range [0, 1], based on this formula:

4 $$s^{\prime} = \frac{s + \alpha }{\beta }$$

where α and β are the scaling parameters compiled by Todeschini et al. [24]. Similarity measures can be categorized according to symmetricity and metricity. A similarity coefficient is called *symmetric* (*S*) if it considers *d* (number of common *off* bits) equally to *a* (number of common *on* bits), *intermediate* (*I*) if *d* is underweighted with respect to *a*, or *asymmetric* (*A*) if *d* is not considered at all. Additionally, the work of Todeschini et al. denotes correlation-based metrics with the letter *Q.* Metricity specifies whether a similarity measure can be transformed into a metric distance, i.e. one that complies with the criteria of non-negativity, identity of indiscernible, symmetry (*d*_A,B_ = *d*_B,A_) and triangle inequality. These can be called (similarity) metrics and are denoted with *M*, while non-metric measures are denoted with *N*. In this work, we have adapted the abbreviations introduced by Todeschini et al. [24].

In our related earlier works, we have confirmed the choice of the Tanimoto coefficient for molecular fingerprints (by a comparison of eight commonly available measures) [26], and more recently we have suggested the *Baroni*–*Urbani*–*Buser* (*BUB*) and *Hawkins*–*Dotson* (*HD*) coefficients for metabolomic fingerprints [25]. We should note however, that due to the highly different data structure, these conclusions are not transferrable to interaction fingerprints (or other fingerprint types).

In this work, our goals were to (1) compare and rank these 44 similarity measures for their use with interaction fingerprint data, and (2) to dissect the interaction fingerprints and investigate how changes in the data structure affect the ranking of similarity coefficients. Also, we aimed to answer some specific questions considering interaction fingerprints, regarding e.g. the usefulness of IFP filtering schemes (i.e. exclusion of certain bit positions or blocks), or of general interaction definitions (e.g. “Any contact”). We note here that we use the abbreviation IFP throughout this work to refer to interaction fingerprints in general, not to the specific fingerprinting method of Marcou and Rognan [6]. (The specific method we used here is a modified version of SIFt [2], implemented in the Schrödinger Suite [4].)

## Methods

### Datasets

Ten protein targets were applied for the comparison, which were selected from the DUD datasets [27] based on the following criteria: (1) a crystal structure of the human protein from the PDB database must be available, (2) the co-crystallized ligand should have a reported bioactivity data (if more structures were available, the one with the most active ligand was selected), and (3) we strived to compile a set of proteins that are as diverse as possible. The applied protein targets and ligand sets are summarized in Table 3.

**Table 3:** Summary of the applied protein targets and ligand sets

	Short name	Name	Uniprot	Protein family	PDB code	No. actives	No. inactives
1	ACE	Angiotensin-converting enzyme	P12821	Hydrolase	4CA5	49	1727
2	ACHE	Acetylcholine esterase	P22303	Hydrolase	4M0F	105	3708
3	ALR2	Aldose reductase	P15121	Oxidoreductase	4XZH	26	917
4	AR	Androgen receptor agonists	P10275	Transcription factor	4OEA	64	2234
5	CDK2	Cyclin dependent kinase 2	P24941	Protein kinase	1AQ1	48	1763
6	COMT	Catechol O-methyltransferase	P21964	Transferase	3BWM	11	428
7	ER	Estrogen receptor antagonists	P03372	Nuclear receptor	3ERT	39	1388
8	PARP	Poly(ADP-ribose) polymerase	P09874	Transferase	4PJT	33	1175
9	SRC	Tyrosine kinase SRC	P12931	Protein kinase	2H8H	155	5784
10	VEGFr2	Vascular endothelial growth factor receptor kinase	P35968	Transferase	3VHE	71	2617

The case studies correspond to ten virtual screening scenarios, where IFPs are used for retrieving the active molecules from among the chemically similar, but not active decoy compounds. A standard tool for evaluating virtual screenings is the area under the receiver operating characteristic curve (ROC AUC, or AUC for even shorter). The AUC can take values between 0 and 1, and corresponds to the probability of ranking a randomly selected active compound higher than a randomly selected inactive compound (as a consequence, an AUC value of 0.5 corresponds to random ranking) [28]. In this work, we have used AUC values as a first approach to evaluating the various IFP-similarity measure combinations, followed by a more detailed statistical analysis, as explained below.

### Generation of interaction fingerprints

All the preprocessing procedures for the protein targets and ligands were carried out with the relevant Schrödinger software (LigPrep, Protein Preparation Wizard etc.) [29]. Standard (default) protocols were used for grid generation and ligand docking (Glide) [30, 31]. The IFPs were also generated with a Schrödinger module based on the docked poses, and contained by default all of the nine interactions listed in Table 1. To study the effects of the more general interaction definitions (bits), we have generated two more sets of IFPs, where we have omitted (1) the Any Contact (Any), and (2) the Any Contact (Any), Backbone Interaction (BB), and Sidechain Interaction (SC) definitions. We have labeled the resulting IFPs ALL (original), WO1 (without Any), WO3 (without Any, BB and SC).

**Fig. 1:**
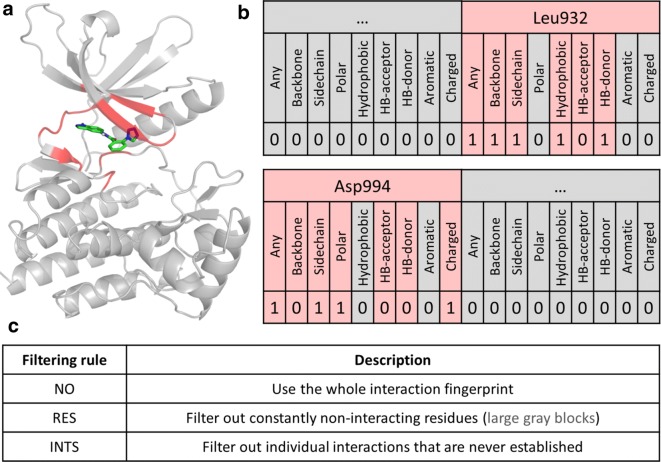
**a** Docked complex of a small-molecule virtual hit (green sticks) to JAK2 [16]. Potentially interacting residues in the vicinity of the ligand are highlighted in red. **b** Excerpt from the interaction fingerprint of the docked complex. Interacting residues are highlighted in red, while non-interacting residues are represented as gray blocks. Inside the red blocks, those interactions are grayed out that cannot be established by definition. **c** Short definition of the SIFt filtering rules implemented in this work. Residue-based filtering (RES) omits any residue that is found to be consistently non-interacting across the whole docked dataset. Interaction-based filtering (INTS) additionally omits any individual interaction that is not established even once across the whole dataset. The latter includes (but is not restricted to) those interactions that cannot be established by definition (grayed-out interactions inside red blocks); for example the “Aromatic” bit will be 0 for any residue that lacks an aromatic ring

Additionally, we have implemented two IFP filtering rules to get rid of the large set of bits in the IFPs, which are consistently 0 across the whole ligand set. Briefly, residue-based filtering (RES) excludes any residue from the IFP that is found to be consistently non-interacting across the whole dataset, while interaction-based filtering (INTS) additionally omits any individual interaction that is never established in the whole dataset. The filtering rules are summarized and illustrated in Fig. 1.

### Similarity metrics

We have implemented a Python module (FPKit) to calculate 44 similarity measures (collected by Todeschini et al. [24]) on plain bitstrings. The definitions of these similarity measures can be found in the original publication of Todeschini et al., and as a supplement to our recent (open access) article on metabolomic profiles [25]. Those measures that do not, by definition, produce values in the [0, 1] range are scaled with the α and β scaling parameters, published together with the definitions (see also Eq. 4). In some instances, we needed to correct some of these scaling parameters and implement additional checks to avoid division-by-zero errors: these are summarized in Additional file 1. The Python module additionally contains the implemented filtering rules, and is available at: https://github.com/davidbajusz/fpkit.

### Statistical analysis

**Fig. 2:**
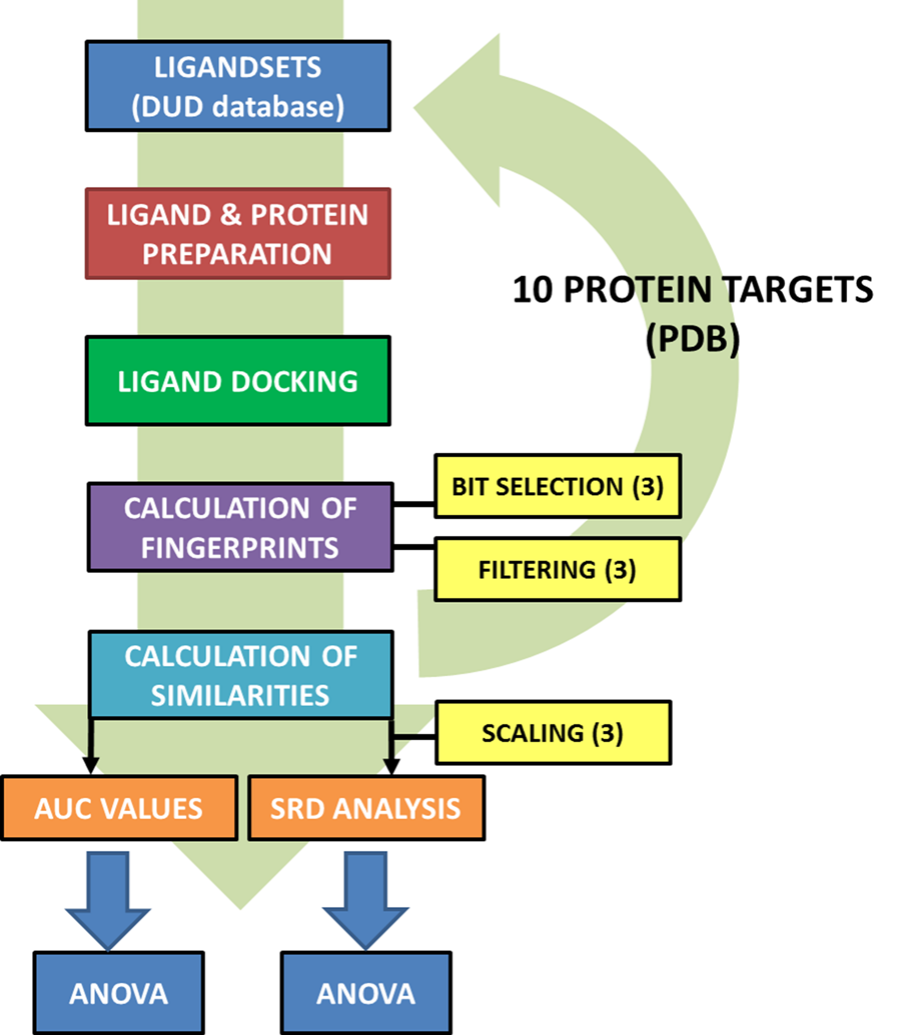
Workflow of the input matrix generation and the complete protocol of the study

Sum of ranking differences (implemented as a Microsoft Excel VBA macro) was used for the evaluation of the similarity values in each of the ten datasets. The similarity measures were scaled with Eq. 4 using the α and β parameters published in [24] (and corrected by us in a few cases, see Additional file 1), but even after scaling, some of the measures produced similarity values in highly different ranges between 0 and 1, therefore additional data pretreatment was used to obtain a balanced set of data, which can be compared in a fair way. The following options were considered for data pretreatment: autoscaling (a.k.a. standardization), rank transformation (i.e. assigning ranks to the values according to increasing magnitude) and range scaling (sometimes wrongly termed interval scaling). The workflow for generating the input matrices for SRD analysis is presented in Fig. 2. Ninety variants of SRD input matrices were calculated based on the different bit selections and filtering rules for each protein target. The input data matrices for SRD analysis contained the similarity values of the molecules, calculated with each of the 44 similarity measures.

SRD is a novel algorithm based on the calculation of the differences between the object-wise ranks produced by a vector (corresponding to a method, model, similarity metric, etc.), as compared to a reference vector [32, 33]. The reference can be experimental values as a gold standard, or a consensus produced by data fusion, such as row-average, minimum or maximum, etc. This is related to the basic idea of multicriteria decision making, where the objective is to rank the objects simultaneously by each criterion: using that terminology, the criteria would be the various similarity measures in this case. The basic steps of the protocol are the following: (1) ranking the samples (here, ligands) in their order of magnitude by each column vector (similarity measure), (2) for each sample (ligand), calculating the differences between the ranks produced by each similarity measure and the reference, and (3) summing up the absolute values of the differences for each similarity measure. The resulting sums are called SRD values and can be used to compare the similarity measures: the smaller the SRD value, the closer the measure is to the reference (in terms of ranking behavior). A detailed animation of the calculation procedure can be found as a supplement to our earlier work [26]. The method is validated with cross-validation and a randomization test as well. The MS Excel SRD macro is freely available for download at: http://aki.ttk.mta.hu/srd

We should note that besides SRD, a number of methods for the comparison of rankings is reported in the literature, or used routinely by statisticians. Spearman’s rank correlation coefficient—probably the most commonly used rank-based statistical test—has been compared to SRD in the paper of Héberger and Kollár-Hunek [34] as early as 2011, and we have also shown in our recent work the more sophisticated discriminatory power of SRD as compared to Spearman’s rho [35]. An interesting novel application of SRD is in Post-Pareto optimality analysis, where it was clearly shown to be a well-suited decision support tool (by ranking the solutions along the Pareto front) [36].

More generally: while it is also based on a comparison of rankings, the SRD workflow can be clearly distinguished from rank-based statistical tests, as it involves not one, but three essential steps. The first of these is the definition of the reference vector (i.e. reference ranking), which—depending of the problem—can be a “gold standard” (such as experimental values for the comparison of computational methods for modeling/predicting the same property) or a consensus of the existing (compared) methods, produced with a suitable data fusion technique, such as average, minimum, maximum, etc. This is again problem-dependent, as the reference vector must always represent a hypothetical optimum (or ideal) ranking. (It may involve more than one data fusion technique, if necessary, e.g. in the present work, the hypothetical best similarity measure would be one that produces the highest possible similarity value for active molecules and the lowest possible value for inactives, so our current solution involved the use of maximum values for actives, and minimum values for inactives, see Results section.) Definition of a reference vector is not part of any rank-based statistical test we are aware of.

The second step is the calculation of the distance measure itself between the reference vector (ranking) and the rankings produced by the compared methods (here, similarity measures). In the current implementation of SRD, the Manhattan distance is applied: in the case where there are no tied ranks, this is identical to another rank-based distance measure, the Spearman footrule metric [37]. Koziol related SRD to another distance measure for permutations—namely, the inversion number [38], but it has less discriminatory power, and has not found any applications yet (to the best of our knowledge).

The third step is the application of a meticulous validation approach, involving a randomization (permutation) test and leave-one-out or leave-many-out cross-validation. This step instantly provides answers to two important questions: whether the SRD values characterizing two compared methods (i.e. rankings) are significantly different from each other (cross-validation), and whether there is any among the compared methods (i.e. rankings) that is not significantly better (i.e. not closer to the reference vector) than random rankings (randomization test).

The further statistical analysis of SRD values was carried out by factorial analysis of variance (ANOVA). This method is based on the comparison of the average values for the different groups of samples. The input matrices contained the SRD values and several grouping factors such as similarity metrics, symmetricity, metricity, bit selection and filtering rule. The complete procedure of statistical analysis was carried out three times with different pretreatment methods (rank transformation, range scaling, autoscaling). STATISTICA 13 (Dell Inc., Tulsa, OK, USA) was used for the analysis.

## Results and discussion

### Comparison based on AUC values

**Fig. 3:**
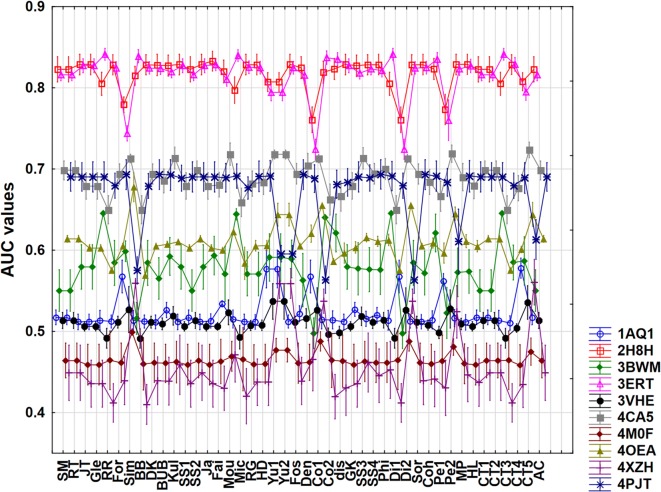
Factorial ANOVA with the use of the protein targets and the similarity measures as factors. (AUC values are plotted against the similarity metrics.) The protein targets (with PDB codes) are marked with different colors and marks on the plot. Average values (dots) and 95% confidence intervals (lines) are shown in each case

As a first strategy, we have used AUC values for the 10 datasets as a basis for comparison and analysis. The AUC values were calculated with the scikit-learn Python package for each dataset and for each of the 44 similarity measures [39]. However, a detailed factorial ANOVA analysis revealed that the AUC values are not fit for the proper evaluation of similarity metrics, because the applied ten protein datasets have very different AUC values, leading to different means and very high standard deviations. In this sense, the AUC values are not sensitive enough to find the most or least consistent similarity measures, when using more than one dataset. Figure 3 illustrates the big differences between the protein targets in terms of AUC values, ranging from excellent classification (2H8H and 3ERT, or SRC kinase and estrogen receptor, respectively) to worse than random classification (4M0F and 4XZH, or acetylcholine esterase and aldose reductase, respectively). There is also no clear consensus regarding the relative performances of the various similarity measures, as the shapes of the curves in Fig. 3 are visibly different (and in some cases display opposite trends).

### Results based on SRD values

Because of the problem detailed above, we have decided to apply the SRD method for the statistical comparison. Selecting the reference value (data fusion) was not trivial in this particular case, since we have active and inactive ligands as well, where the ideal behavior for a similarity measure is to produce the highest and the smallest similarity values, respectively. Thus, the reference was defined as the minimum or maximum value among the similarity values, depending on the activity of the specific ligand (if it was active, the row-maximum was used, if it was inactive, then row-minimum was used). The analysis was run 90 times altogether, corresponding to each possible combination of 10 protein targets, 3 bit selections, and 3 filtering rules.

The original input matrices contained the 44 similarity measures for the different molecules in each case study, but the ranges of these measures were sometimes very different. For example, values close to 0 were typical for the Mou (Mountford) similarity, but values close to 1 were typical for the Yu1 (Yule) similarity. Obviously, in such cases, taking the row minimum as the reference value would favor the former, regardless of the ligand being active or inactive. Thus, an additional round of data pretreatment was essential for the analysis, to provide a valid basis of comparison. Autoscaling, range scaling and rank transformation were applied for this purpose.

One example of the original plots produced by the SRD script can be seen in Additional file 1: Figure S1, where the normalized (scaled) SRD values are plotted in increasing magnitude and the distribution of random SRD rankings (for random numbers) is plotted as a basis of comparison.

SRD analysis was performed with fivefold cross-validation to every combination of the original parameters (bit selection, filtering, scaling) and the results (the SRD values) for each similarity measure were collected from every dataset (see Fig. 2) for a final factorial ANOVA analysis. The collected SRD values for the ten datasets (i.e. target proteins) were used together for the further ANOVA analysis, to allow us more general conclusions.

**Fig. 4:**
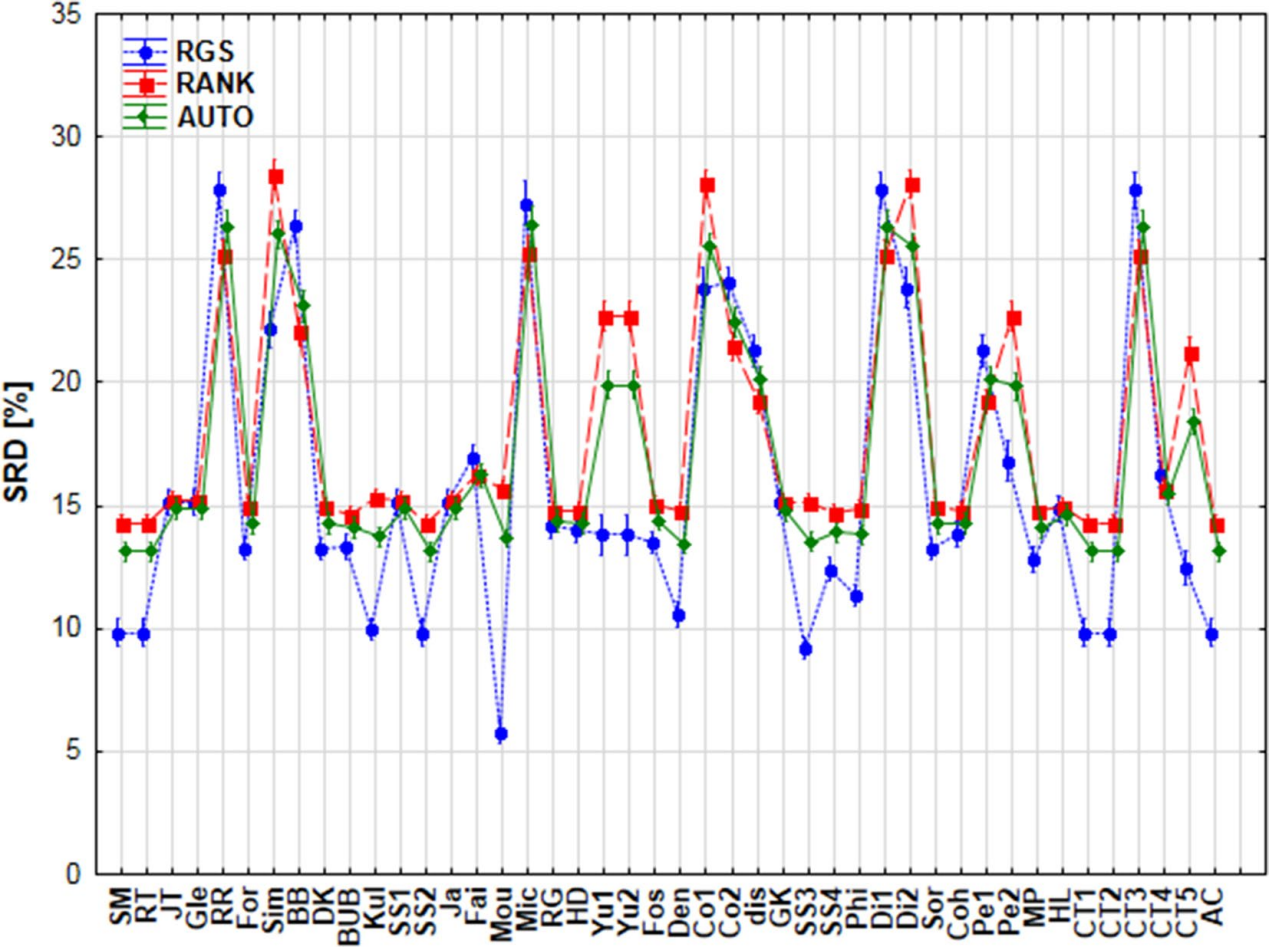
Factorial ANOVA with the use of scaling and similarity metrics as factors. Normalized SRD values [%] are plotted against the similarity metrics. The different scaling methods are marked with different symbols and lines. (RGS: range scaling, RANK: rank transformation, AUTO: autoscaling.)

First, we have compared the data pretreatment methods, to select a suitable one for the rest of the analyses. The effect of pretreatment was significant according to ANOVA, meaning that the results were significantly different for the different scaling options, as seen in Fig. 4. For the further analyses, we have chosen to use autoscaling, as range scaling and rank transformation are more biased and more sensitive to outliers. Additionally, autoscaling can be considered as a consensual choice between the other two (see Fig. 4).

**Fig. 5:**
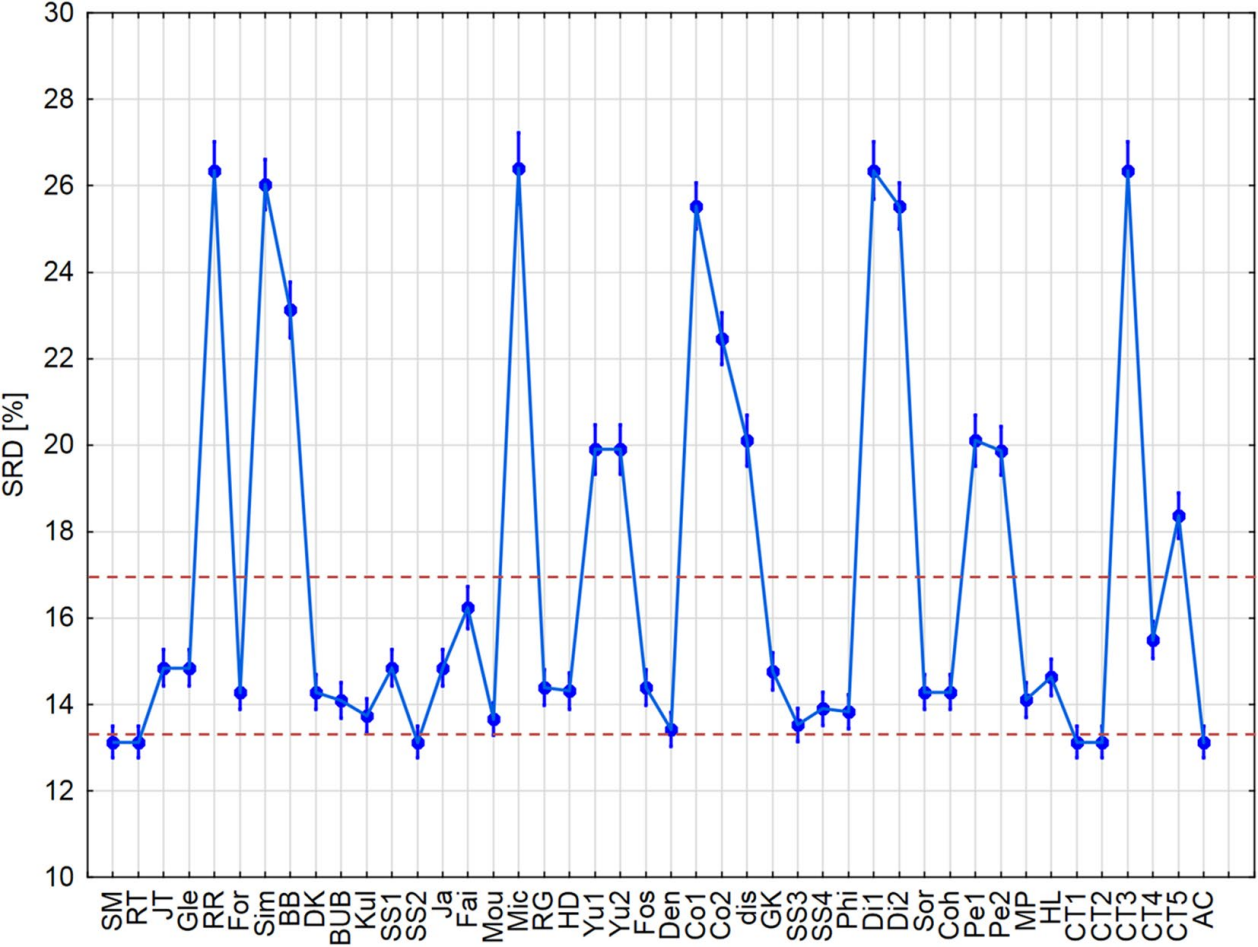
Factorial ANOVA with the similarity measures as the factor. Average values are marked with blue dots and the blue lines below and above the dots denote 95% confidence intervals. Normalized SRD values [%] are plotted against the similarity measures. The red dashed lines are arbitrary thresholds defined to select the best few metrics, and to identify the region with the less consistent similarity measures

From this point on, standardized data were used for the further ANOVA analyses. The input matrix contained a total of 23,760 rows, corresponding to SRD values for each possible combination of 44 similarity measures, three filtering rules, three types of bit selections, six cross-validation rounds (fivefold cross-validation, including one round using the whole dataset, “All”), and ten datasets. (Part of the input matrix can be seen in Additional file 1: Table S1 for the better understanding of the ANOVA procedure.) We examined all the possible factors: similarity measures (44), bit selections (3), filtering rules (3), symmetricity (4) and metricity (2). With the use of these dependent factors, we can conclude whether their effects (one by one, or in combination) were significant on the α = 0.05 level based on the normalized SRD values. In the case of similarity measures the final outcome can be seen in Fig. 5.

We can observe that there are some measures with very high SRD values (i.e. producing very different rankings as compared to the reference/consensus method), for example RR (Russel–Rao), Mic (Michael) or CT3 (Consonni–Todeschini 3). On the other hand, one can identify the best measures (i.e. closest to the reference) as SM (simple matching) [40], RT (Rogers–Tanimoto) [41], SS2 (Sokal–Sneath 2) [42], CT1 (Consonni–Todeschini 1), CT2 (Consonni–Todeschini 2) [43] or AC (Austin–Colwell) [44]. These similarity measures are closer to the reference and can be recommended for usage. The JT (Jaccard–Tanimoto) metric, which is the de facto standard of cheminformatics (simply called the “Tanimoto coefficient” in most of the related scientific literature) is located relatively close to the reference, but somewhat farther than those mentioned above, meaning that the SM, RT, SS2, CT1, CT2 and AC metrics could be considered as viable alternatives of the Tanimoto coefficient.

**Fig. 6:**
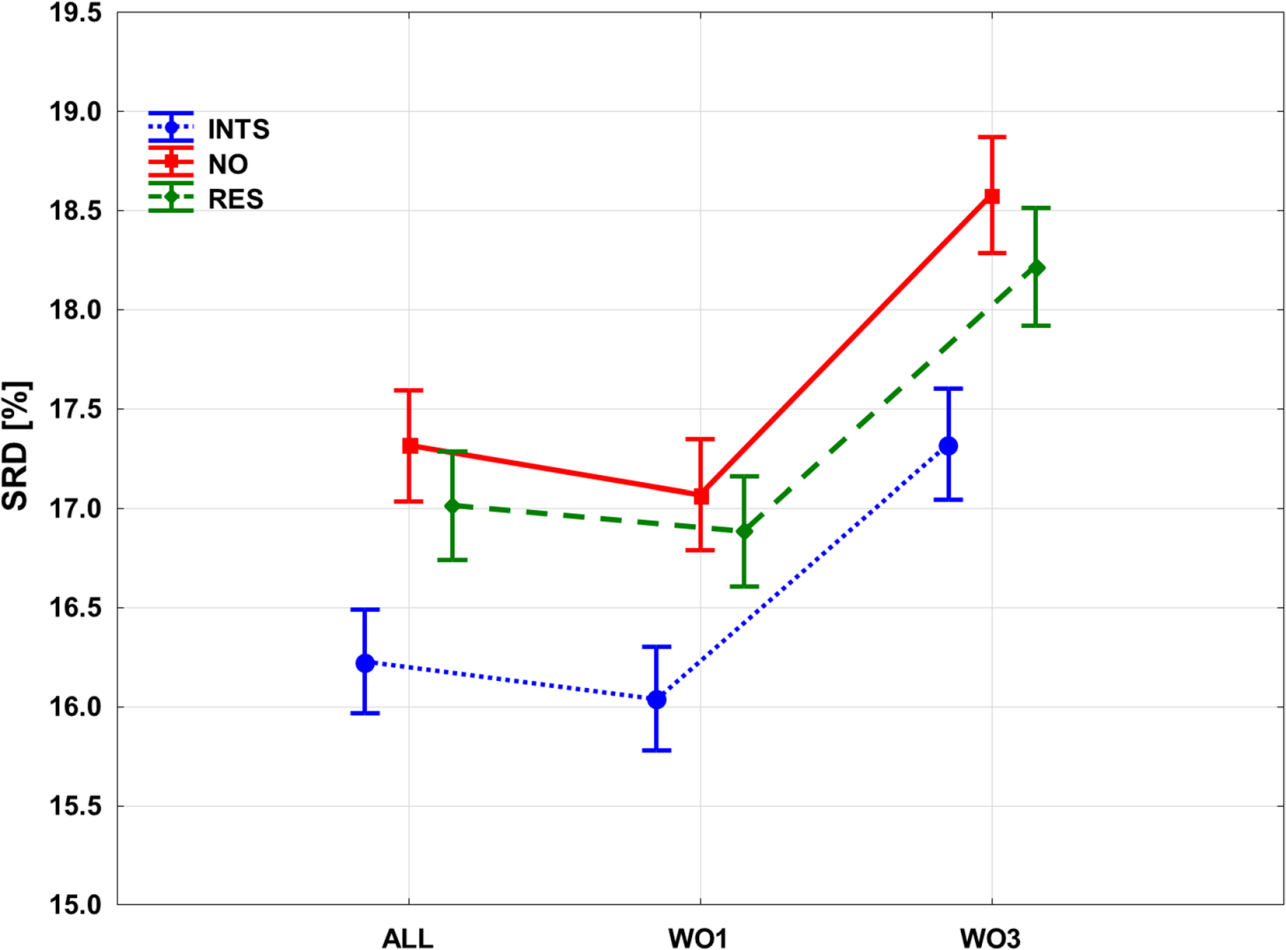
Factorial ANOVA with the bit selection and the filtering rule as dependent factors. SRD values [%] are plotted against the bit selection options. Interaction based filtering (INTS) is marked with a blue dotted line, no filtering (NO) is marked with a red continuous line and residue based filtering (RES) is marked with a green dashed line

If we examine the effects of the bit selection and filtering rule together, the ANOVA plot can be seen in Fig. 6. Significant differences between the filtering methods and the bit selections can be clearly observed. Interaction-based filtering (INTS) clearly improves the results, and so does residue-based filtering (RES) to a smaller extent. The differences between the bit selections are also clear: omitting the “Any contact” bit (WO1), results in a slight, but significant improvement, but omitting the BB and SC bits (Backbone and Sidechain interactions) causes a serious deterioration of SRD values. In summary, the best combination is the use of interaction-based filtering (INTS), while omitting the “Any contact” bit.

**Fig. 7:**
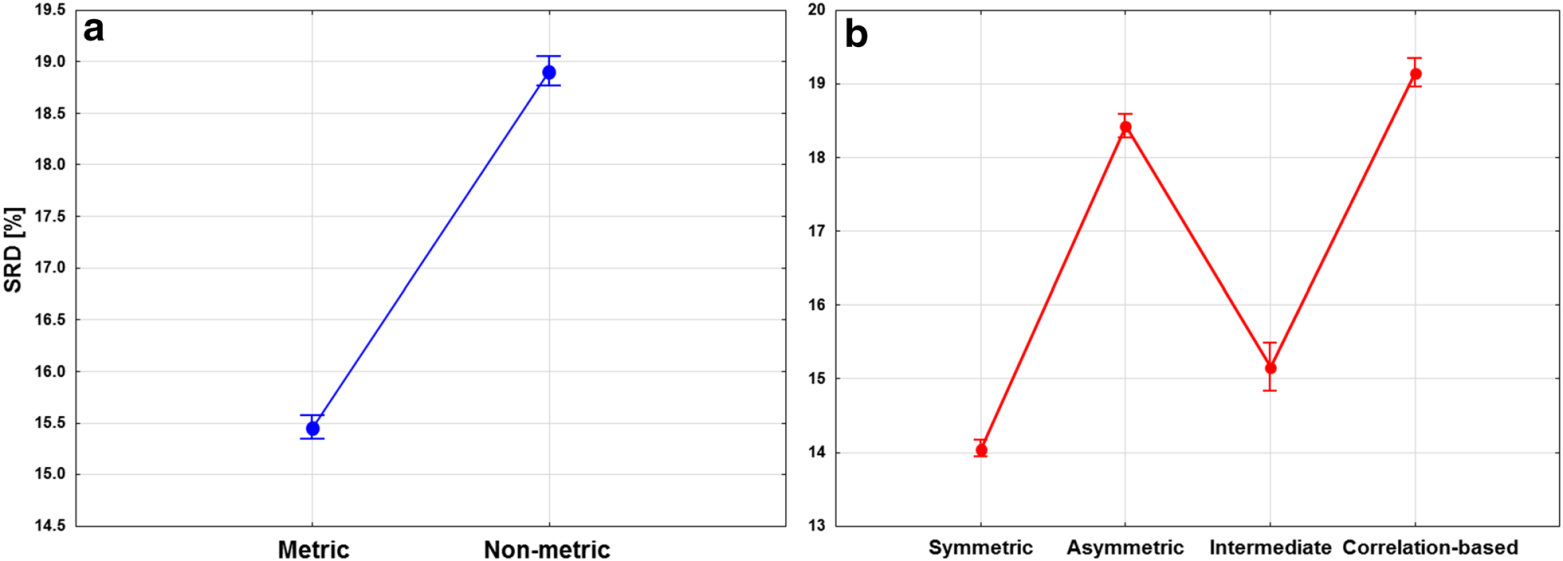
The result of ANOVA analysis with metricity (**a**) and symmetricity (**b**) as factors. SRD values [%] are plotted against the different groups of similarity measures. Average values are plotted and the 95% confidence intervals are indicated with whiskers

The similarity measures can be grouped by symmetricity and metricity (see Introduction). ANOVA plots based on these factors are included in Fig. 7. It is clearly seen on Fig. 7a that metric similarity measures give, on average, much closer results to the ideal reference method than non-metric measures. According to Fig. 7b, symmetric and intermediately symmetric similarity measures tend to give more consistent results with the reference method. Both factors gave statistically significant differences (at α = 0.05) between the groups.

The preference for symmetric measures over asymmetric ones is somewhat surprising, considering that one would expect symmetric measures to be affected by the amount of “off” bits (and consequently, the number of common “off” bits, *d*) more than asymmetric ones. If we look at the effects of the filtering rules (and therefore the amount of “off” bits) on the SRD values of the similarity metrics separately (Additional file 1: Figure S2), we find that this assumption is confirmed, but only partially: similarity measures, where we can observe major differences are Mic (Michael), HD (Hawkins–Dotson), Den (Dennis), dis (dispersion), SS4 (Sokal–Sneath 4), Phi (Pearson–Heron), Coh (Cohen), Pe1, Pe2 (Peirce), MP (Maxwell–Pilliner), and HL (Harris–Lahey). These are symmetric and correlation-based coefficients, without exception. The associated ANOVA plots are included in Additional file 1: Figure S2.

## Summary and conclusion

In this study forty-four similarity measures were compared based on ten case studies, corresponding to interaction fingerprint-based virtual screening scenarios. The effects of the applied set of bits (interaction types) and filtering rules were studied in detail. The comparison was carried out with a novel algorithm, sum of ranking differences (SRD), coupled with analysis of variance (ANOVA). This work complements our earlier comparative studies on metabolomic fingerprints [25] and molecular fingerprints [26].

There are several similarity metrics that are worth consideration as viable alternatives of the popular Jaccard–Tanimoto coefficient, namely: Sokal–Michener (SM), Rogers–Tanimoto (RT), Sokal–Sneath 2 (SS2), Consonni–Todeschini 1 and 2 (CT1, CT2) and Austin–Colwell (AC). These six similarity measures gave the most consistent results with the “ideal” (hypothetical best) reference method in our evaluations using 10 highly diverse protein data sets. We can also conclude that metric similarities are usually more consistent with the reference method than non-metric ones. Similarly, symmetric and intermediately symmetric measures gave more consistent results than asymmetric and correlation-based ones.

Finally, there are important and significant differences with regard to the applied bit definitions and filtering rules. As a general conclusion, we can recommend omitting the “Any contact” bit definition from IFP-based analyses, as it will not deteriorate the results in a virtual screening scenario (however, omitting the backbone and sidechain interaction bits, BB and SC, is not recommended). Similarly, applying a bit filtering rule, such as interaction-based filtering (omitting any interaction that is not established even once in the whole dataset) can improve the results on average. The open-source Python-based FPKit (FingerPrint Kit) package applied for IFP filtering and similarity calculations is freely available at: https://github.com/davidbajusz/fpkit.

## List of abbreviations

The abbreviations and definitions of similarity metrics can be found in the work of Todeschini et al. [24] and our recent open access article on metabolomics fingerprints [25].

### Bit selections

ALL: all interactions; WO1: all interactions, except “Any contact”; WO3: all interactions, except “Any contact”, “Backbone interaction” and “Sidechain interaction”.

### Filtering rules

INTS: interaction-based filtering; NO: no filtering; RES: residue-based filtering.

### Statistical methods

ANOVA: analysis of variance; SRD: sum of (absolute) ranking differences.

### Data pretreatment

AUTO: autoscaling (or standardization); RGS: range (interval) scaling; RANK: rank transformation.

## Additional file

**Additional file 1. ** Supplementary information.
