# Supplementary material for: Life beyond the Tanimoto coefficient: similarity measures for interaction fingerprints
Source: J Cheminform. 2018 Oct 4;10:48. doi: 10.1186/s13321-018-0302-y (PMC6755604; doi:10.1186/s13321-018-0302-y)
Supplement: Supplementary file 1 — Additional file 1. Supplementary information. [file 13321_2018_302_MOESM1_ESM.docx]

**SUPPLEMENTARY INFORMATION**

**Life beyond the Tanimoto coefficient: similarity measures for interaction fingerprints**

Anita Rácz^1^, Dávid Bajusz^2,*^, Károly Héberger^1^

^1^ *Plasma Chemistry Research Group, Research Centre for Natural Sciences*,

*Hungarian Academy of Sciences, H-1117 Budapest, Magyar tudósok krt. 2, Hungary*;

^2^ *Medicinal Chemistry Research Group, Research Centre for Natural Sciences,*

*Hungarian Academy of Sciences, H-1117 Budapest, Magyar tudósok krt. 2, Hungary*

**Table of contents**

1. Adjustments made to the similarity measure definitions 2
2. Supplementary tables and figures 3
3. References 6

* To whom correspondence should be addressed:

Dávid Bajusz

E-mail: bajusz.david@ttk.mta.hu

**1. Adjustments made to the similarity measure definitions**

Corrections to scaling factors:

- the scaling factors of the CT5 (Consonni-Todeschini 5) metric have been corrected to α = +1 and β = 2
- the scaling factor β of the Den (Dennis) metric was corrected to β = $3\frac{\sqrt{p}}{2}$
- the scaling factor β of the Sco (Scott) metric has been corrected to β = 2 (this measure was not included in the present work as it was identified to give identical results to the RG, or Rogot-Goldberg metric by Todeschini *et al.* [1])

Checks to avoid division-by-zero (in addition to those published by Todeschini *et al.* [1]):

- SS3 (Sokal-Sneath 3): if any of the four addends would produce a division by zero, than that part is set to zero (the other parts are unaffected)
$s_{SS3}=\frac{1}{4}\left(\frac{a}{a+b}+\frac{a}{a+c}+\frac{d}{b+d}+\frac{d}{c+d}\right)$
- Pe1 and Pe2 (Peirce 1 and 2): if the denominator would be zero, the similarity is set to zero (becomes 0.5 when scaled)
$s_{Pe1}=\frac{ad-bc}{(a+b)(c+d)}$
$s_{Pe2}=\frac{ad-bc}{(a+c)(b+d)}$

- CT4 (Consonni-Todeschini 4): if the denominator would be zero, the similarity is set to zero (stays 0 when scaled)
$s_{CT4}=\frac{\ln(1+a)}{\ln(1+a+b+c)}$

**2. Supplementary tables and figures**

**Figure S1.** One of the original plots produced by the SRD script (3VHE protein, WO3 bit selection and RES filtering rule) [2, 3]. Normalized SRD values [%] are plotted on the X and left Y axes (thus, the distance from the reference vector is represented by the column heights and the column distances from the origin as well). Cumulative relative frequencies of SRD values in the case of random rankings can be seen on the right Y axis. The plot on the bottom is a magnified version of the one on top.


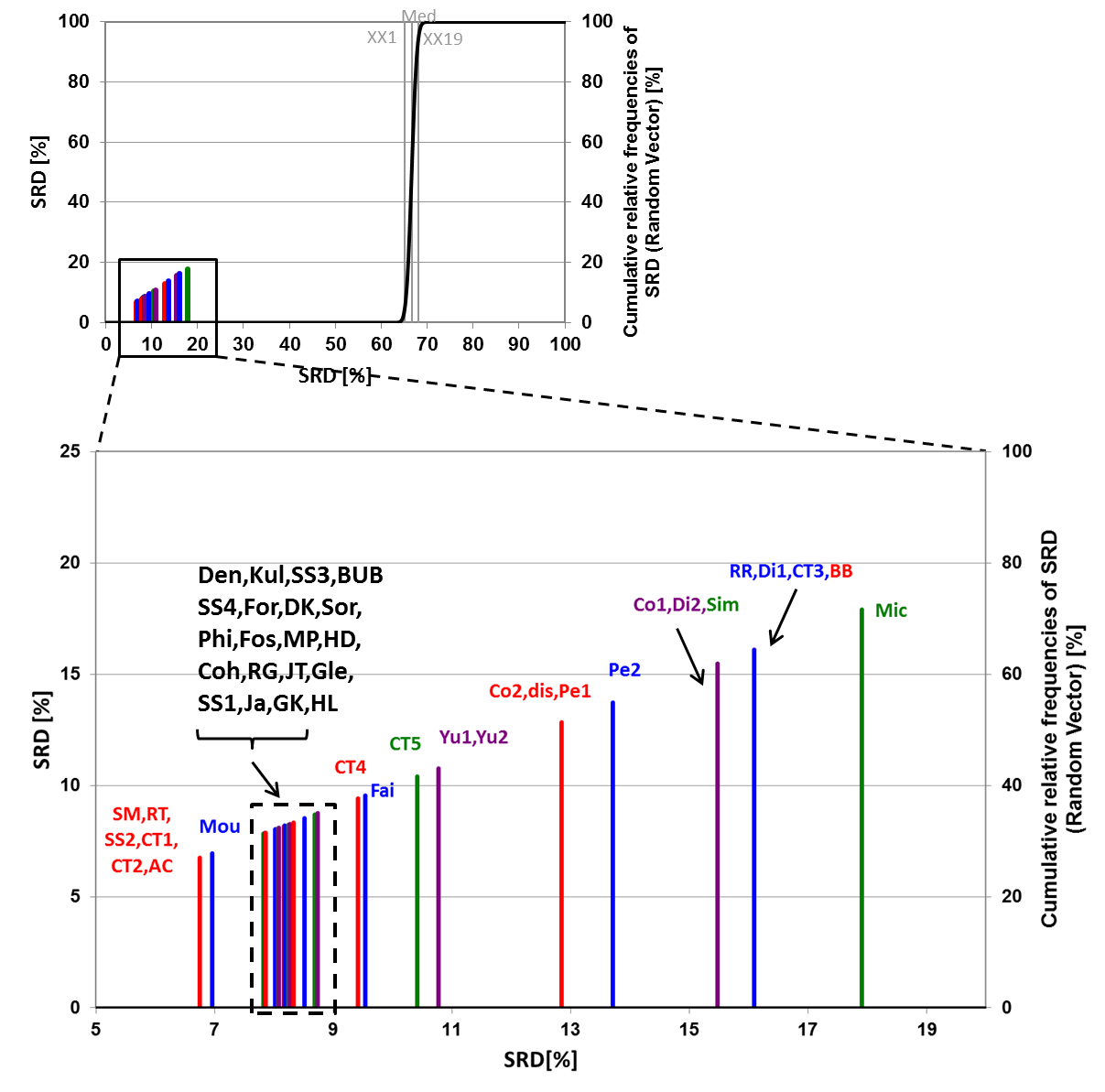


**Table S1.** A part of the standardized data matrix for ANOVA analyses. Protein, Bits, Filtering, Metric (similarity measures), Symmetricity and Metricity were indicated as factors for ANOVA. The independent variable was the SRD values for the comparison.


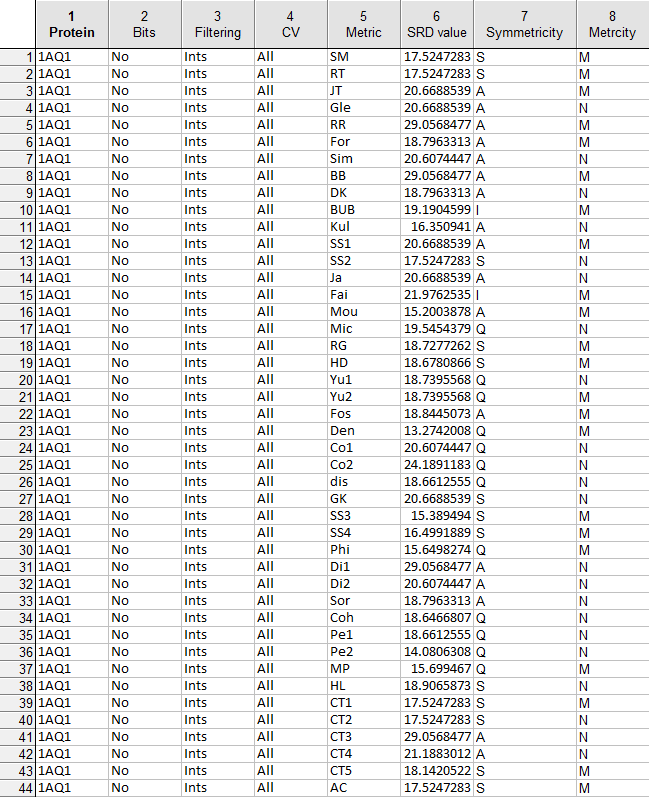


**Figure S2.** Summary of the ANOVA results for the 44 similarity measures with the factor of the different filtering rules. The significantly differing measures are framed in red. The normalized SRD values are on the *y* axis and the filtering rules are in the following order on the *x* axis in each plot: INTS, NO, RES. (See next page.)

**
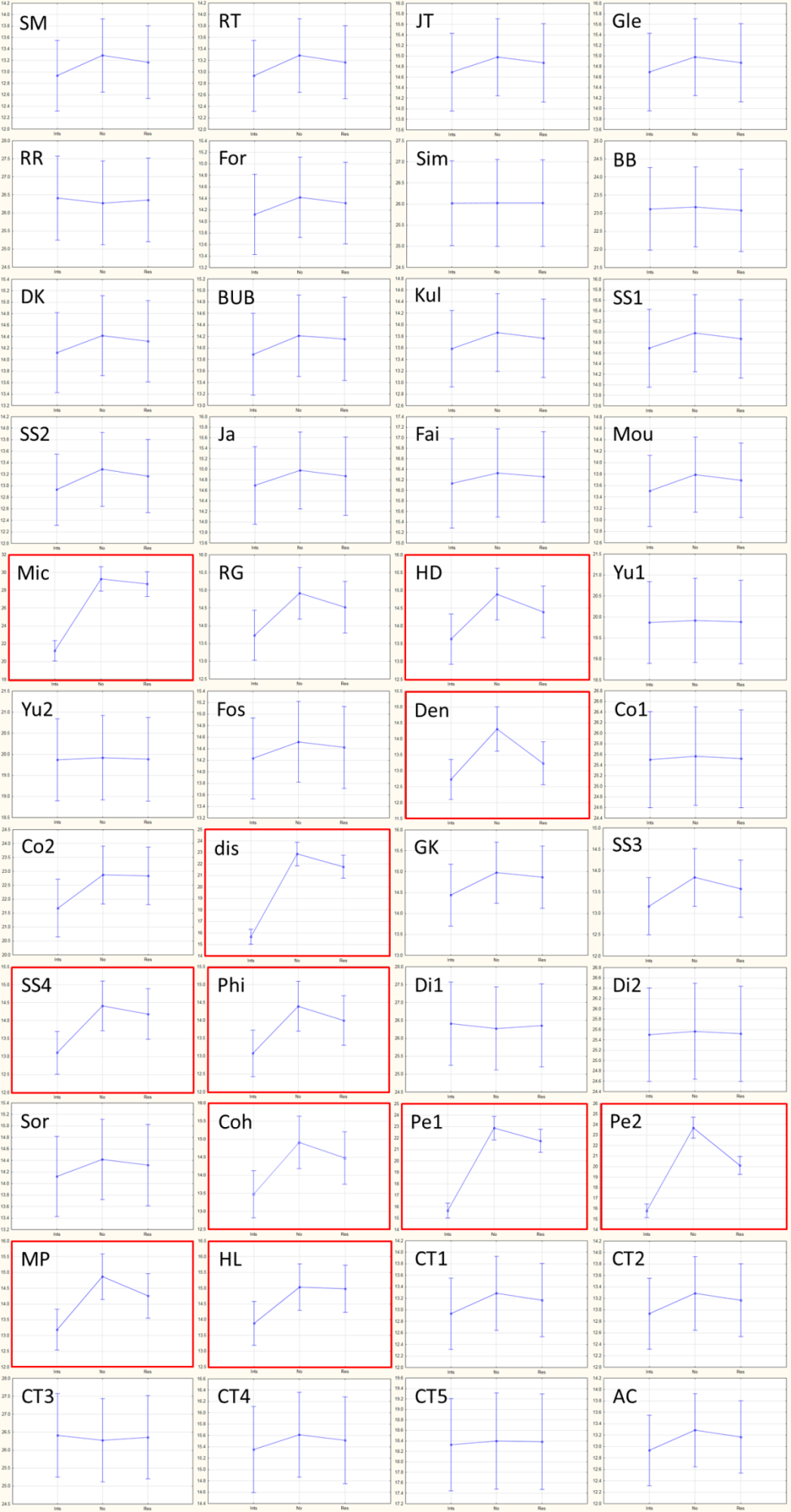
**

**References**

1. Todeschini R, Consonni V, Xiang H, et al (2012) Similarity coefficients for binary chemoinformatics data: overview and extended comparison using simulated and real data sets. J Chem Inf Model 52:2884–2901

2. Héberger K (2010) Sum of ranking differences compares methods or models fairly. TrAC Trends Anal Chem 29:101–109 . doi: 10.1016/j.trac.2009.09.009

3. Kollár-Hunek K, Héberger K (2013) Method and model comparison by sum of ranking differences in cases of repeated observations (ties). Chemom Intell Lab Syst 127:139–146 . doi: 10.1016/j.chemolab.2013.06.007
